# In vitro evaluation of chloroquine-loaded and heparin surface-functionalized solid lipid nanoparticles

**DOI:** 10.1186/s12936-018-2302-9

**Published:** 2018-04-02

**Authors:** Joseph O. Muga, Jeremiah W. Gathirwa, Matshawandile Tukulula, Walter G. Z. O. Jura

**Affiliations:** 1 Maseno University, Post Office Box 333, Maseno, Kisumu-Busia Road, Kisumu, 40105 Kenya; 2 Kenya Medical Research Institute, Nairobi, Kenya; 3 Polymers and Composites, Material Sciences and Manufacturing, Council for Scientific and Industrial Research (CSIR), Pretoria, 0001 South Africa; 4 Department of Chemistry, Tshwane University of Technology, Pretoria, 0001 South Africa

**Keywords:** Heparin, Chloroquine-encapsulated, Solid lipid nanoparticle, Antiplasmodial activity

## Abstract

**Background:**

Use of chloroquine, an otherwise safe and relatively affordable anti-malarial drug, was discontinued due to widespread prevalence of resistant parasites. Many entrant anti-malarial drugs for treatment of chloroquine resistant malaria raises the concerns of cost and safety among other challenges. Innovative ways of circumventing chloroquine resistance is of paramount importance. Such may include nanoparticulate delivery strategies and targeting. This study evaluated physicochemical properties and in vitro antiplasmodial activity of chloroquine encapsulated heparin functionalized solid lipid nanoparticles (CQ-Hep-SLNs) and non-heparin functionalized SLNs (CQ-SLN) against *Plasmodium falciparum*.

**Methods:**

The modified double-emulsion solvent evaporation technique was used to prepare the nanoparticles. HPLC/UV was used to determine the in vitro drug release. The semi-automated micro-dilution technique was adapted in assessing the in vitro antiplasmodial activity to give drug concentration capable of inhibiting 50% of the *P. falciparum* (IC_50_), as a function of antiplasmodial efficacy.

**Results:**

Prepared nanoparticles were below 500 nm in size with % drug loading (%DL) between 21 and 25% and encapsulation efficiency (%EE) of 78–90%. The drug-loaded SLN exhibited a biphasic drug release profile at pH 7.4, with an initial burst release during the first 24 h followed by sustained release in both formulations. Nanoformulated CQ-SLN (4.72 ± 0.14 ng/mL) and CQ-Hep-SLN (2.41 ± 0.27 ng/mL), showed enhanced in vitro antiplasmodial activities against chloroquine sensitive (D6) strain of *P. falciparum,* albeit with no activity against the chloroquine resistant W2 strain, compared to free CQ standard (5.81 ± 0.18 ng/mL).

**Conclusions:**

These findings suggest that the nanoformulated drugs displayed enhanced anti-malarial activities against chloroquine sensitive (D6) strains of *P. falciparum* compared to the free CQ standard. There is some form of potential dual synergistic effect of CQ-loaded heparinized solid lipid nanoparticles (Hep-SLN), meaning that combining heparin and CQ in SLNs has beneficial effects, including potential for specific targeting of parasitized red blood cells as afforded by heparin. Thus, the study has produced SLNs nanoparticles that have superior in vitro activities than CQ on CQ-sensitive parasites.

## Background

Malaria is a public health problem in more than 90 countries, inhabited by a total of some 2.4 billion people or about 40% of the world’s population [1, 2]. Mortality due to malaria stood at 429,000 deaths worldwide in 2015, with 90% of these occurring in sub-Saharan Africa and the majority being children under 5 years of age [2]. In Kenya, malaria is the leading cause of morbidity and mortality where more than 70% of the population live in malaria risk areas and accounting for nearly 15% of all out-patient attendance in the health facilities admissions and 10% of hospital admissions [3–5]. The high morbidity and mortality in sub-Saharan Africa are due to *Plasmodium falciparum*, the most virulent of the five malarial parasites that infect humans. Successful malaria elimination is still a challenge in the absence of new vaccines, drugs and vector control strategies. This challenge, more especially in terms of chemotherapy and vaccine development, is attributed to the complex life cycle of the parasite where it is able to hide, propagate itself and transfer itself between hosts [6]. In the absence of an effective vaccine, chemotherapy is the only option readily available for managing malaria. Much of this morbidity and mortality could be avoided if drugs available to patients were efficacious, of high quality and used correctly [7]. Chloroquine (CQ) was, for several decades, the anti-malarial drug of choice due to its safety, high efficacy and low cost [8]. However, due to the widespread prevalence of chloroquine-resistant (CQR) parasite strains, CQ was replaced as the front-line anti-malarial chemotherapy in the late 1990s. Artemisinins are the most potent compounds in the anti-malarial drug arsenal and no suitable replacements are expected any time soon [7]; artemisinin-based combination therapy (ACT) is currently the recommended first-line treatment of uncomplicated falciparum malaria [9]. A report of resistance to ACT in South East Asia has further complicated malaria control efforts [10]. Increased efforts in anti-malarial drug discovery are, therefore, urgently needed but, in the absence of new drugs, drug reformulation, more specifically drug delivery, seems an attractive option [11, 12]. One possible strategy is to reformulate the available anti-malarials in an innovative way and nanotechnology has emerged as the best way of delivering the drugs to target site while potentially mitigating resistance [13, 14].

Targeted drug delivery systems can provide an increased drug bioavailability and selectivity in achieving the intake of total amounts of drugs sufficiently low to not be harmful to patients but high enough to kill the parasite. Recent studies by Fernandez-Busquets and co-workers demonstrated that heparin-functionalized liposomal nanocarriers can selectively deliver the drug cargo to parasitized red blood cells (pRBCs) over non-infected ones (uRBCs) [15–18]. Incorporation of heparin to nanoparticles showed improved stealth capable of bypassing clearance by the reticuloendothelial system, improved targeting of molecules with enhances uptake and accumulation and increased stability and solubility [19]. Furthermore, over and above targeting, heparin also provides a dual action to the drugs because of its inherent anti-malarial activity [18]. Notwithstanding the fact that liposomes have an advantage of mimicking biological systems, their use has been associated with various shortcomings such as poor stability, “leakage” and delivering very small quantities of drugs in the target site to have any noticeable biological effect [20, 21]. Thus, the current study proposes to reformulate CQ in more robust heparin-surfaced functionalized solid-lipid nanoparticles (SLNs) and evaluate the in vitro against chloroquine sensitive and chloroquine resistant *P. falciparum* strains.

## Methods

### Materials

All materials and reagents were purchased from commercial sources and used as received without any modifications; CQ diphosphate salt (analytical grade), stearic acid, low viscous chitosan (CS), polyvinyl alcohol (PVA) of molecular weight 13,000–23,000 partially hydrolyzed (87–89%), d-lactose monohydrate, sulfanoyl, ethyl acetate (EtOAc)and low molecular weight heparin sodium salt (> 180 USP units/mg) were purchased from Sigma Aldrich and/or Merck (both in South Africa and Kenya). Purification of the solutions of CS, polyvinyl alcohol (PVA and d-lactose monohydrate was undertaken via membrane filtration. Acetonitrile and trimethylamine used as mobile phase in high-performance liquid chromatography (HPLC) (SHIMADZU, Shimadzu Corporation, Japan) were purchased from Merck. Deionized water was obtained from the Barnstead EasyPure (II) UV-ultrapure water system (Thermo Fisher Scientific, USA) and was used throughout the study. Magnetic stirrer and hot plate with max rpm of 6000, high speed homogenizer with max rpm of 8000 (Silverson L4R; Silverson Machines Limited, Buckinghamshire, UK), bench top Bucchi mini spray dryer (model B-290; BUCHI Labortechnik AG, Flawil, Switzerland), and UV–VIS instrument (Perkin Elma) were used in nanoformulation.

### Preparation of empty and CQ-loaded SLNs

The modified double-emulsion solvent evaporation technique was adapted, with slight modification, from the method previously reported [22, 23]. Briefly, 2 mL of aqueous PVA (2%, w/v) solution and 10 mL EtOAC containing 100 mg of stearic acid were homogenized at 6000 rpm, in an ice-bath, for a period of 5 min to form the first emulsion (w_1_/o). This emulsion was then transferred into a second aqueous solution (containing 10 mL PVA (2%), 5 mL of 0.3% chitosan solution (w/v), 5 mL of 5% d-lactose monohydrate solution and 5 mL of 1% heparin solution) allowed to stir on a magnetic stirrer for 2 min, followed by addition of 200 µL of sulfanoyl. Thereafter, the resultant mixture was homogenized at 8000 rpm for 5 min to produce water-in-oil-in water (w_1_/o/w_2_) double emulsion which was directly fed into Buchi mini spray dryer set to the following parameters: Outlet temperatures (90 °C, Aspirator = 100%, pump = 2 mL/min and atomizing pressure of 7 bars) to afford an off white amorphous powder in excellent yields (> 90%). In the case of drug loaded SLN, 100 mg CQ diphosphate salt was dissolved in the first 2 mL aqueous PVA solution and all other steps used for the empty SLNs were followed to yield CQ loaded SLN also in excellent yields (> 90%).

### Characterization of the SLNs

Particle size, polydispersity index (PDI) and zeta potential were measured using the dynamic laser scattering or photon correlation spectroscopy (Malvern Zetasizer Nano ZS, Malvern instruments, Malvern, UK) with measurements conducted at 25 °C and at an angle of 173°. Approximately 1 mg of the dried SLN was suspended in 1 mL of de-ionized water, vortexed and sonicated before measuring. The intensity-weighted mean value was determined and the average of three measurements taken.

### Characterization of the drug loading, encapsulation efficiency and in vitro drug release

The percentage drug-loading (%DL) and encapsulation efficiency (%EE) were measured using a UV–VIS spectroscopy via the indirect method and calculated as follows:

The EE % and DL % was calculated using the formulas below:

1 $${\text{EE}}\% = \left( {{\text{drug in precipitate}}/{\text{total added drug}}} \right) \times 100$$

2 $${\text{DL}}\% =\, \left( {{\text{drug in precipitate}}/{\text{drug in precipitate}} + {\text{added excipients}}} \right) \times 100$$

Where, “drug in precipitate” = total drug added-free drug after ultra-centrifugation (indirect method) and “added excipients” = lipids + surfactant mixtures + other ingredients used.

The amount of chloroquine (CQ) released from the solid lipid nanoparticles (SLNs and Hep-SLN) was determined in triplicate using HPLC/UV [24]. A Gemini-NX reverse-phase C18 column (250 mm × 4.60 mm internal diameter, pore size of 5 µm; Phenomenex, CA 90501-1430, USA) was used. The mobile phase consisted of a mixture of acetonitrile and 0.1% trimethylamine and was delivered at a flow rate of 0.5 mL/min for a period of 5 min per run. Detection of the column effluent was conducted at 260 nm. To characterize drug release, CQ-loaded SLNs (1 mg/mL in Eppendorf tubes) were incubated in phosphate saline buffer pH 7.4 at 37 °C in a shaking bath (100 rpm) over 120 h. At set time points, the SLNs were removed from the bath, centrifuged at 5000 rpm for 5 min, supernatant collected and analysed for CQ concentration using the HPLC method described in reference 24. Samples were diluted with acetonitrile before injection, in order to match the HPLC mobile phase.

### In vitro antiplasmodial evaluation

The Sierra Leonean chloroquine-sensitive (CQS)D6 and the Indochinese chloroquine-resistant (CQR) W2 strains were used for the in vitro study. The parasite clones were obtained from the Malaria Research and Reference Reagent Resource Center (MR4) and cultured at the Kenya Medical Research Institute (KEMRI) in Nairobi Kenya.

Parasite cultivation was carried out using previously described procedures [25, 26]. The culture medium consisted of RPMI 1640 (10.4 g/L) powdered medium (without PABA) and lactic acid (LA) dissolved in 960 mL of distilled-autoclaved water (DAW) supplemented with 10% human serum, 25 mM (5.94 g/L) HEPES and 25 mM NaHCO_3_. Human O+ve red blood cells served as the parasites host cells. Test samples (SLN, CQ-SLN, Hep-SLN and CQ-Hep-SLN) solutions were prepared in 100% DMSO (Sigma Chemical Co, St Louis, MO, USA) which was diluted to lower the concentration of DMSO, to ≤ 1% to avoid solvent carry over effects, leading to an effective concentration of 80 ng/mL (CQ + excipients) for the samples. Chloroquine base was prepared for use as a reference drug. The semi-automated micro-dilution technique was adapted in assessing in vitro antiplasmodial activity [27]. Briefly, 96-well flat-bottom micro-culture plates were pre-coated with test solutions in duplicate. The first row of wells (A), served as controls. Thus, wells 1–8 of row H served as negative controls (parasitized and no drug) while wells 9–12 of row H served as a background (non parasitized and no drug). Row B contained test solutions of the drug at the highest concentration. Serial dilution was carried out under sterile conditions in a laminar flow hood (Bellco Glass Inc., U.S.A) using a Titertek motorized hand diluter (Flow Laboratories, Uxbridge, UK) from the second (B) to the last well (H) achieving a 64-fold dilution. Parasite cultures at ≥ 80% ring-stage, ≥ 4% percentage parasitaemia (%P), and ≥ 3% growth rate and at a 6% haematocrit were used in the antiplasmodial assays. Growth media was used to adjust this culture prior to introducing into the wells so as to achieve a parasitaemia of 0.4% and a haematocrit of 1.5% from which 200 µL were dispensed into each well of the drug pre-coated micro-culture plate. Plates containing parasitized and non-parasitized erythrocytes were incubated at 37 °C in a gas mixture (3% CO_2_, 5% O_2_, and 92% N_2_) for 48 h after which 25 µL of 0.5 mCi (G-3H) hypoxanthine (Amersham International, Burkinghamshire, UK) in culture medium was added to each well followed by further 18 h incubation. At the end of the incubation the assay plates were frozen and thawed to lyse the cultures. The parasite DNA was recovered by harvesting the lysate onto glass-fibre filter plates using cell harvester and the radioactivity in counts per minute (CPMs) determined using a beta counter (Wallac Micro Beta TriLux). The mean values for uptake of 3H-hypoxanthine in parasitized control and non parasitized control erythrocytes were calculated. The drug concentration capable of inhibiting 50% of the *P. falciparum* (IC_50_) was determined by logarithmic transformation of drug concentration and CPMs using the formula:

$$IC_{50} = antilog \, \left( {log \, X_{1} + \left[ {\left( {logY_{50} {-} \, log \, Y_{1} } \right) \, \times \, \left( {log \, X_{2} {-} \, log \, X_{1} } \right)} \right]/\left( {logY_{2} {-}log \, Y_{1} } \right)} \right)$$

where Y_50_ is the CPM value midway between parasitized and non-parasitized control cultures and X_1_, Y_1_, X_2_, and Y_2_ are the concentrations and CPM values for the data points above and below the CPM midpoints [28].

### Statistical analysis

The in vitro antiplasmodial activity of the encapsulated CQ was expressed by the inhibitory concentrations (IC_50_) of the drug that induced 50% reduction in parasitaemia compared to the negative control (100% parasitaemia).

## Results and discussions

### Characterization of the SLNs

The physicochemical characteristics of all the prepared nanoparticles are shown in Table 1. The empty SLN had a significantly bigger size (482.2 ± 12.0 nm) and zeta potential (24.0 ± 0.321 mV) compared to all the other prepared nanoparticles. This trend was expected since the empty SLN lack the electrostatic interaction expected between the positively charged chitosan and the negatively charged species (CQ-diphosphate and heparin), hence the bigger size and the large positive surface charge. As anticipated, encapsulation of CQ resulted in nanoparticles reduced in size and the surface charge. Further reduction in size and surface charge was observed upon addition of the highly negatively charged heparin as seen with both Hep-SLN and CQ-Hep-SLN. Not surprisingly, the SLNs that contained both the CQ and heparin were the smallest in size (374.6 ± 7.6 nm). The drug loading percentage (%DL: 25%) and encapsulation efficiency (%EE: 90%) for the surface unfunctionalized SLN were slightly higher compared to the heparin functionalized SLN (%DL: 21% and %EE: 78%). The small difference in the %DL and %EE could be attributed to the repulsive effect of the CQ and heparin, leading to these being lower when the two are present.

**Table 1:** Physicochemical characteristics of SLN and Hep-SLN (empty and drug-loaded)

DRUG	Size(nm) (Mean ± std dev.)	PDI (Mean ± std dev.)	Zeta (mV) (Mean ± std dev.)	Drug-loading (%)	EE (%)
SLN	482.2 ± 12.0	0.245 ± 0.023	24.0 ± 0.321	–	–
SLN-CQ	444.5 ± 6.9	0.175 ± 0.021	9.41 ± 0.376	25	90
SLN-HEP	379.2 ± 1.2	0.346 ± 0.028	− 5.73 ± 0.267	–	–
SLN-HEP-CQ	374.6 ± 7.6	0.272 ± 0.053	− 4.06 ± 0.091	21	78

### In vitro drug release profiles

**Fig. 1:**
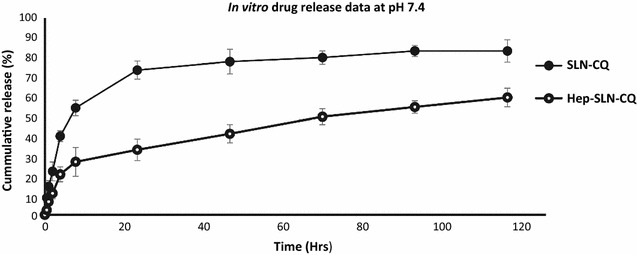
Cumulative in vitro release profile of CQ from SLN and Hep-SLN in phosphate buffer saline (PBS)(pH 7.4) at 37 °C, over a 120 h period

A characteristic biphasic release profile for both CQ-loaded SLN and Hep-SLN was observed and an initial burst release phase (occurring up to 24 h, and representing CQ release of up to 76% for SLN compared to only 33% of Hep-SLN), followed by a sustained release phase (Fig. 1). Drug release was tracked up to 120 h and at this time point 83 and 60% of encapsulated CQ had been released by the SLN and Hep-SLN, respectively. Hep-SLN released significantly less drug than the SLN at both 24 and 120 h, suggesting that the incorporation of the heparin enhanced the sustained release properties of the Hep-SLN.

### In vitro antiplasmodial evaluation of the SLNs

The in vitro antiplasmodial evaluation results of the empty SLN, CQ-loaded SLN (CQ-SLN: without surface functionalization), heparin-surface functionalized SLN (Hep-SLN: empty surface functionalized SLN without CQ) and CQ-loaded surface functionalized SLN (CQ-Hep-SLN) are all shown in Table 2. The data shown in Table 2 includes SLNs activities based on (i) gross SLNs weights, (ii) actual CQ amounts at specific drug loadings and (iii) on actual CQ amounts at specific drug loadings and in vitro drug release data during the assay time (66 h). CQ diphosphate salt (CQ) and heparin sodium salt (Heparin Na salt) were used as standards. Surprisingly, although heparin is known to possess inherent antiplasmodial activity [18], in this study no activity was observed for the pure heparin sodium salt (> 100 µg/mL) on both strains at the tested concentrations.

**Table 2:** *In vitro* antiplasmodial activity of the prepared SLNs

Samples	D6 IC_50_ (based on gross weight of SLNs)	D6 IC_50_ (based on actual CQ concentration at 25 and 21% DL for CQ-SLN and CQ-Hep-SLN)	D6 IC_50_ (Based on actual CQ concentration at 25 and 21% DL and in vitro drug release at 66 h for CQ-SLN (80%) and CQ-Hep-SLN (50%)	W2 IC_50_
Heparin Na salt	> 100 µg/mL	–	–	> 100 µg/mL
SLN	> 100 µg/mL	–	–	> 100 µg/mL
CQ-SLN	23.75 ± 3.24 ng/mL	5.94 ± 0.18 ng/mL	4.752 ± 0.144 ng/mL	> 400 ng/mL
Hep-SLN	12.98 ± 1.62 µg/mL		–	> 100 µg/mL
CQ-Hep-SLN	22.97 ± 2.58 ng/mL	4.82 ± 0.54 ng/mL	2.41 ± 0.27 ng/mL	> 400 ng/mL
CQ drug	5.81 ± 0.18 ng/mL	5.81 ± 0.18 ng/mL	5.81 ± 0.18 ng/mL	58.71 ± 2.67 ng/mL

All the SLNs exhibited enhanced antiplasmodial activity compared to the standard CQ drug on CQ-sensitive D6 strain but no activity against CQ-resistant W2 strain. The in vitro antiplasmodial bioassay was based on the [3H]hypoxanthine incorporation method [25]. In this method, the parasites are initially exposed to the test samples over 48 h after which tritiated hypoxanthine is added followed by further 18 h incubation. Thus, the malaria parasites are exposed to the test samples for a total of 66 h. In this study the in vitro CQ release from the SLNs indicated that only about 50 and 80% of CQ had been released and exposed to the parasite for CQ-Hep-SLN and CQ-SLN, respectively, based on the 66-h test cycle. Extrapolating the actual amount of CQ exposed to the parasite in the used in vitro bioassay model, IC_50_ would thus be 4.752 ± 0.144 ng/mL and 2.41 ± 0.27 ng/mL for CQ-SLN and CQ-Hep-SLN, respectively. This would translate to about 50% improved antiplasmodial activity for CQ-Hep-SLN in comparison to free CQ drug whose IC_50_ was 5.81 ± 0.18 ng/mL, while CQ-SLN has comparable activity to free CQ. This data further points to some form of a synergistic effect brought about by combining heparin and CQ in SLNs, including the specific binding expected of Hep-SLNs to pRBCs as a result of heparin, due to the fact that CQ-Hep-SLN had better activity than CQ-SLN. The implication of this data is that with SLNs, a steady drug exposure to the parasite can be maintained over a lengthy period of time. This can significantly reduce the need for frequent dosing in malaria treatment, reduce dose amount, thus reduce cost and toxicity and at the same time improve patient compliance.

## Conclusion

The findings show that both CQ-SLN and CQ-Hep-SLN had enhanced in vitro antiplasmodial activities against CQS D6 strain compared to free CQ standard but no effect against CQR W2 strain of *P. falciparum*. Specifically, CQ-Hep-SLN showed about 50% more in vitro efficacy, while CQ-SLN had comparable but better efficacy than the free standard CQ. These findings further suggests some form of dual synergistic effect brought about by combining heparin and CQ in SLNs and this has the potential of further being exploited in order to restore to potency of anti-malarial drugs due to the specific targeting provided by heparin. Thus, this strategy can further be used to deliver poorly soluble drugs, such as lumefantrine for example, to the desired target sites in small enough quantities required to kill the parasite. Planned future work will involve undertaking in vivo efficacy and pharmacokinetic studies on these formulations, as well as an extensive optimization a later stage.

## Abbreviations

ACT: artemisinin-based combination therapy
CQ: chloroquine diphosphate
CQS: chloroquine sensitive
CQR: chloroquine resistant
CS: chitosan
SLN: solid lipid nanoparticle
CQ-SLN: chloroquine-solid lipid nanoparticle
Hep-SLN: heparin-solid lipid nanoparticle
CQ-Hep-SLN: chloroquine-heparin-solid lipid nanoparticle
*p*RBC: parasitized red blood cell (cells infected with malaria parasite)
PVA: polyvinyl alcohol (surfactant)
*u*RBCs: red blood cell not infected with malaria parasite

## References

[1] Date AA, Joshi MD, Patravale VB (2007). Parasitic diseases: liposomes and polymeric nanoparticles versus lipid nanoparticles. Adv Drug Deliv Rev.

[2] WHO (2016). World malaria report 2016.

[3] Maina JK, Macharia PM, Ouma PO, Snow RW, Okiro EA (2017). Coverage of routine reporting on malaria parasitological testing in Kenya, 2015–2016. Global Health Action.

[4] USAID, President’s Malaria Initiative (2016). Kenya Malaria Operational Plan FY.

[5] Afrane YA, Zhou G, Githeko AK, Yan G (2014). Clinical malaria case definition and malaria attributable fraction in the highlands of western Kenya. Malar J.

[6] Santos-Magalhães NS, Mosqueira VCF (2010). Nanotechnology applied to the treatment of malaria. Adv Drug Deliv Rev.

[7] WHO (2014). World malaria report 2014.

[8] Nayyar GM, Breman JG, Newton PN, Herrington J (2012). Poor-quality antimalarial drugs in southeast Asia and sub-Saharan Africa. Lancet Infect Dis.

[9] Hyde JE (2007). Drug-resistant malaria—an insight. FEBS J.

[10] WHO (2017). World malaria report 2017.

[11] De Jong WH, Borm PJ (2008). Drug delivery and nanoparticles: application and hazards. Int J Nanomed.

[12] Yan L, Chen X, Tjong S-C (2013). Nanometerial for drug delivery. Nanocrystalline materials: their application-structure-property relationships and application.

[13] Yan Y, Yang Y, Zhang W, Chen X (2014). Advanced materials and nanotechnology for drug delivery. Adv Mater.

[14] Singh R, Lillard JW (2009). Nanoparticle-based targeted drug delivery. Exp Mol Phathol.

[15] Urbán P, Fernàndez-Busquets X (2014). Nanomedicine against malaria. Curr Med Chem.

[16] Marques J, Vilanova E, Mourão PAS, Fernàndez-Busquets X (2016). Marine organism sulfated polysaccharides exhibiting significant antimalarial activity and inhibition of red blood cell invasion by *Plasmodium*. Sci Rep.

[17] Marques J, Moles E, Urbán P, Prohens R, Busquets MA, Sevrin C (2014). Application of heparin as dual agent with antimalarial and liposome targeting activties towards *Plasmodium*-infected red blood cells. Nanomedicine.

[18] Aláez-Versón CR, Lantero E, Fernàndez-Busquets X (2017). Heparin: new life for an old drug. Nanomedicine.

[19] Passirani C, Barrat G, Devissaguet JP, Labarre D (1998). Interactions of nanoparticles bearing heparin or dextran covalently bound to poly(methyl methacrylate) with the complement system. Life Sci.

[20] Sercombe L, Veerati T, Moheimani F, Wu SY, Sood AK, Hua S (2015). Advances and challenges of liposome assisted drug delivery. Frontiers Pharmacol.

[21] Zhang L, Granick S (2006). How to stabilize phospholipid liposomes (Using nanoparticles). Nano Lett.

[22] Omwoyo WN, Melariri P, Gathirwa JW, Olo F, Mahanga GM, Kalombo L (2016). Development, characterization and antimalarial efficacy of dihydroartemisinin loaded solid lipid nanoparticles. Nanomedicine.

[23] Omwoyo WN, Ogutu B, Olo F, Swai H, Kalombo L, Melariri P, Mahanga GM (2014). Preparation, characterization, and optimization of primaquine-loaded solid lipid nanoparticles. Int J Nanomedicine.

[24] Miranda TA, Silva PHR, Pianetti GA, César IC (2015). Simultaneous quantitation of chloroquine and primaquine by UPLC-DAD and comparison with HPLC-DAD method. Malar J.

[25] Trager W, Jensen JB (1976). Human malaria parasites in continuous culture. Science.

[26] Moll K, Ljungström I, Perlmann H, Scherf A, Wahlgren M (2013). Methods in malaria research.

[27] Desjardins RE, Canfield CJ, Haynes JD, Chulay JD (1979). Quantitative assessment of antimalarial activity in vitro by a semiautomated microdilution technique. Antimicrob Agents Chemother.

[28] Sixsmith DG, Watkins WM, Chulay JD, Spencer HC (1984). In vitro antimalarial activity of tetrahydrofolate dehydrogenase inhibitors. Am J Trop Med Hyg.

